# Proposing a novel ABCDEF framework for managing critical illness in geriatrics: challenges and perspectives

**DOI:** 10.1080/07853890.2025.2579797

**Published:** 2025-10-28

**Authors:** Jiacheng Shen, Jing Yan, Jianhong Xie, Li Li

**Affiliations:** a Geriatric Medicine Center, Department of Geriatric Medicine, Zhejiang Provincial People’s Hospital (Affiliated People’s Hospital, Hangzhou Medical College), Hangzhou, China; b Department of Critical Care Medicine, Zhejiang Hospital, Hangzhou, China

**Keywords:** Critical illness, geriatrics, patient-centered care

## Abstract

**Background:**

As the global population ages rapidly, the number of older adults experiencing critical illness is increasing significantly. This growing demographic presents substantial challenges for critical care systems worldwide. However, older patients are frequently underrepresented in clinical research and often receive suboptimal care due to assumptions about low productivity, the presence of multiple comorbidities, polypharmacy, and altered physiological responses to illness and treatment. These factors contribute to a paucity of targeted studies and evidence-based guidelines for managing critical illness in geriatric populations. In contrast to younger patients, older adults often place greater emphasis on long-term outcomes and quality of life rather than survival alone. As a result, the clinical management of critical illness in this population involves distinct and complex considerations.

**Methods:**

We propose the adoption of a practical and scalable ABCDEF framework: A, Artificial Intelligence; B, Value-Based Medicine; C, Comprehensive Care Planning; D, Doctor–Patient Shared Decision-Making; E, Enhanced Recovery for Surgical and Critically Ill Patients; F, Frailty Assessment and Early Identification of High-Risk Older Adults. This framework is our core contribution, designed specifically to integrate key geriatric principles into the high-intensity setting of critical care in a structured and actionable manner.

**Conclusions:**

The ABCDEF framework offers a structured, holistic approach to geriatric critical care that improves patient-centered outcomes, optimizes resource utilization, and addresses existing gaps in research and clinical practice, ultimately enabling more compassionate, effective, and sustainable care delivery. We advocate for this framework as a proactive strategy to transform the standard of care for a growing and vulnerable patient population, moving beyond mere survival towards meaningful recovery.

## KEY MESSAGES

1. The growing older adult population represents a significant challenge for the field of critical care.
2. There remains a notable lack of systematic research and evidence-based guidance specifically tailored to the management of critical illness in geriatrics.
3. We propose the adoption of a practical and scalable ABCDEF framework to address these challenges.

## Introduction

The global demographic landscape is undergoing rapid changes, with China being home to the largest geriatric population cohort. In 2023, the proportion of the population aged 60 and above is 21.1%, with expectations soaring above 30% by 2035. The number of older person aged 80 years and above will reach 250 million, with expectations soaring to 17% by 2035 [1]. This demographic shift correlates with a notable surge in geriatric patients admissions to intensive care units (ICUs), accentuating the escalating demand for intensive care services [2,3].

Aging leads to a decline in organ functional reserve and reduced tolerance to physiological stressors. Concurrently, compensatory mechanisms progressively deteriorate with advancing age, contributing to chronic inflammation and metabolic dysregulation [4]. The cardiopulmonary system is particularly vulnerable to these age-related changes. In the heart, aging is characterized by cardiomyocyte loss, ventricular wall thickening, reduced cardiac output, and associated immunometabolic disturbances accompanied by increased inflammatory activity [5]. Pulmonary aging involves decreased airway macrophage density, diminished lung elastic recoil, and impaired alveolar gas exchange efficiency, which elevate the risk of respiratory infections and compromise systemic oxygenation [6,7]. Immunosenescence further disrupts host defense mechanisms, thereby increasing susceptibility to infections, malignancies, and autoimmune disorders [4]. The growing population of older adults and the clinical complexities of aging present significant challenges in critical care [8].

Managing of critical illness in geriatrics is inherently complex, involving not only medical care but also ethical, social, and organizational challenges. A key aspect is the need for humanized care that maintains patient dignity and autonomy, especially in high-pressure settings like emergency and prehospital care. For example, ambulance personnel must balance life-saving interventions with respecting the values and experiences of geriatric patients [9].

Training inadequacies further complicate care. A study on caring for children with serious illness reveal significant gaps in training for palliative and end-of-life care, issues that are equally relevant for geriatrics [10]. Effective management of critical illness in the geriatrics requires training that addresses multiple comorbidities, pain management, cognitive impairments, and end-of-life decisions.

Post-discharge rehabilitation is another critical area, where geriatric patients often struggle due to complex health conditions and limited self-management abilities. Remote Health Monitoring (RHM) technology, utilizing wearable devices to collect physiological data, shows promise in enhancing rehabilitation for geriatric patients recovering from critical illnesses [11].

However, most clinical trials in critical care focus on younger populations due to societal priorities that view younger populations as having higher potential for recovery and productivity [12]. In contrast, geriatrics presents with multiple comorbidities, polypharmacy, and altered physiological responses to both illness and treatment [13].

These complicate clinical decision-making, as healthcare providers must meticulously balance treatment risks and benefits to mitigate adverse outcomes such as medication interactions, delirium, and prolonged hospital stays. Effectively addressing critical illnesses in the geriatric patients remains a perplexing quandary [14]. This gap highlights the urgent need for a comprehensive management framework with critical illness in geriatrics.

## Discussion

For geriatric patients, the ultimate objective of intensive care treatment is to achieve long-term survival with the high quality of life, especially for critical illnesses in patients over 80, who face limited life expectancy due to their age. For many geriatric patients, the foremost concern isn’t death itself but rather becoming dependent.

Therefore, When an critically ill older patient is presented for admission to the ICU, key clinical data is needed to rapidly assess the patient’s short and long-term prognosis and determine the appropriateness of ICU admission. These data [15], in addition to demographics, reason for admission, severity of illness, also include health status prior to hospital admission, usually before the acute deterioration (14 days prior to admission frequently used), frailty status, cognitive decline, activities of daily living and comorbidities exercise tolerance, etc. The incidence of comorbidities (such as hypertension, diabetes, chronic obstructive pulmonary disease, etc.) also need to be considered [16].

The physiology of aging and available evidence are applied, and patients and their families are included in the scope of diagnosis and treatment [17]. In addition, risk factors for diseases that may affect the quality of life of critically ill geriatrics need to be considered [18]. Nonetheless, practical challenges are associated with provision of cares for geriatric patients as highlighted below.

Advanced age often accompanies a multitude of comorbidities, predisposing patients to rapid disease fluctuations. Instances such as recurrent acute illness, rapid disease progression, multi-organ dysfunction, and the development of persistent inflammation, immunosuppression, and catabolism syndrome (PICS) are frequent occurrences [19].

In addition, factors such as drug–drug interactions, malnutrition, cognitive impairment, decreased metabolic rates, sarcopenia, immunosenescence, as well as declining cardiac and respiratory reserves, significantly influence therapeutic decisions and care management in the geriatric patients.

Finally, acute geriatric patients often present with nonspecific complaints, lacking distinctive clinical manifestations, thereby heightening the risk of misdiagnosis or underdiagnosis. Additionally, prevalent cognitive impairments among the older patients affect the reliability of self-reported quality of life, further complicating care provision [20].

Therefore, managing geriatric patients in acute and emergency situations necessitates specific strategies focusing on ‘risk assessment’ and ‘step down’. Within the ICU settings, emphasizing ‘adjustment and balance’, practicing ‘moderation and compensation’, preventing decompensation, and averting Iatrogenic injury are imperative [21]. Early targeted assessments and effective interventions addressing common critical organ-related issues in the geriatric patients require meticulous attention.

With the emergence of new medications and innovative technologies, a forward-looking care framework are urgently needed to be tailored specifically for critical illness in geriatrics. We propose the adoption of a straightforward and implementable ABCDEF framework (Table 1).

**Table 1.** The ABCDEF framework for managing critical illness in geriatrics.

Component	Key elements
A: Artificial Intelligence (AI)	Integrates multidimensional data, including geriatric syndromes, biological age, and clinical acuity, into intelligent diagnostic and therapeutic decision-making. Facilitates seamless interconnection and interoperability of patient information across healthcare institutions. Provides evidence-based guidance for therapeutic strategies, comorbidity and polypharmacy management, and prediction of adverse outcomes. Enhances frailty assessments, enables early identification of high-risk patients, and supports the development of individualized care plans.
B: Value-based medicine	Focuses on maximizing health outcomes relative to the cost incurred, thereby enhancing cost-effectiveness in healthcare delivery. Shifts the paradigm from disease-centered to health-centered care by incorporating broader determinants of health and considering the overall societal impact. Emphasizes the integration of healthcare services, multi-level health protection strategies, and comprehensive evaluation of outcomes across the entire care continuum.
C: Comprehensive Care Plan	Encompasses the entire continuum of care across diverse settings, including home, community, nursing home, emergency department, ICU, post-ICU transition, and rehabilitation. Establishes comprehensive, time-sequenced data records to ensure seamless care continuity. Utilizes compact home-based monitoring devices to facilitate post-discharge follow-up and enable robust data aggregation. Aligns clinical management with patient-centered goals by prioritizing functional recovery and long-term prognosis.
D: Doctor–Patient Shared Decision-Making (SDM)	Actively engages patients and their surrogates in medical decision-making by systematically integrating individual preferences, values, and clinical evidence. Employs time-limited trials (TLT) in settings of prognostic uncertainty to facilitate a structured and time-bound evaluation of therapeutic response. Incorporates artificial intelligence to enhance documentation efficiency and support accurate information interpretation, thereby promoting more consistent, transparent, and high-quality SDM.
E: Enhanced Recovery for Surgical or Critically illness	Introduces expedited rehabilitation interventions to mitigate the risk of delirium, venous thromboembolism (VTE), sarcopenia, and other prevalent geriatric complications. Leverages geriatric interdisciplinary teams, such as Acute Care for Elders (ACE) units and Geriatric Evaluation and Management (GEM) programs, to deliver integrated, patient-centered care. Implements structured co-management models integrating geriatricians and surgical specialists to optimize perioperative care and enhance postoperative outcomes.
F: Frailty Assessment and Early Identification	Requires routine frailty assessment for all older adults at ICU admission using validated instruments such as the Clinical Frailty Scale (CFS) and the Frailty Index (FI). Utilizes emerging digital tools, including the electronic Frailty Index (eFI), to enable real-time monitoring and dynamic risk stratification. Promotes structured multidisciplinary collaboration between intensivists and geriatricians to ensure comprehensive evaluation and integrated care planning. Integrates AI to enhance the consistency and reliability of frailty scoring, supporting early identification of high-risk patients and informing individualized care strategies.

### Artificial intelligence (AI)

AI technology is used to integrate multi-dimensional comprehensive information including geriatric syndrome, biological age, acute disease severity, combined with doctor–patient joint decision-making, to provide intelligent and accurate diagnosis and treatment decisions for critical illness in geriatrics. AI will help to realize the interconnection and interoperability of patient information among multi-level medical institutions. Superior medical institutions provide remote assistance to geriatric patients with acute or critically ill conditions in primary medical institutions to ensure the safety of the transitional care and provide optimal monitoring and timely treatment.

In addition, AI can support clinical decision-making by guiding therapy strategies, managing multiple chronic conditions, ensuring the safe use of polypharmacy, and predicting adverse clinical events [22–24]. Specifically, AI can play a crucial role in developing individualized care plans (C), identifying high-risk patients (C), improving doctor–patient communication (D), optimizing comorbidity management (E), and enabling more accurate frailty assessments (F).

However, several issues warrant careful consideration when applying AI to critical illness in geriatrics. First, AI models must be externally validated specifically in cohorts of older adults with critical illness and undergo targeted fine-tuning or transfer learning to mitigate performance degradation in the very old and frail [23]. Second, clinician-facing human–AI interfaces should be systematically designed to optimize the cognitive workflow between clinicians and AI systems, thereby reducing risks associated with both over-reliance and under-reliance on algorithmic support [24]. Finally, robust safeguards for patient privacy are imperative; data storage and access must strictly comply with applicable legal and regulatory requirements [23,24].

## Value-based medicine

Value-based medicine refers to a medical management model in which healthcare institutions monitor the medical efficacy of a specific patient group, analyzes the resources and costs required to achieve expected effects, obtains medical effects per unit currency, and takes the optimal value as the goal to continuously improve the treatment strategy [25]. The essence of value medicine is patient-centered, aiming to obtain the best treatment effect with the least cost [26].

Value-based medicine focuses on the factors that affect health, shifting from disease-centered to health-centered, from focusing on the treatment of existing diseases to the treatment of non-disease, from relying only on the health system to the overall society [21]. Value-based medicine pays more attention to the integrity, coordination and realizability of people’s health, pays more attention to integrated medical and health services in service provision, pays more attention to multi-level health protection in medical insurance services, and pays more attention to the full cycle effect evaluation in medical services.

ICU treatments incur significant costs. During the treatment of critical illness in geriatrics, it needs to consider the quality of medical care and the cost performance of medical services. In the future, it might be prudent to formulate guidelines and consensus rooted in the paradigm of value-based medicine, complementing traditional evidence-based approaches. While the term ‘Value-based Medicine’ may seem broad, its core principle—maximizing health outcomes per unit of cost—is paramount in resource-intensive critical care for older patients.

## Comprehensive care plan

Managing critical illness in geriatrics involves a comprehensive continuum of care that spans home-based settings, community-supported services, nursing homes, emergency departments, triage, ICUs, post-ICU care, rehabilitation facilities, and family involvement, among other components. During ICU admission, patients require serial assessments of functional status, disability-free survival, health-related quality of life, cognitive performance, and delirium, as well as prognostic indicators such as expected duration of mechanical ventilation, ICU length of stay, mortality risk, discharge destination, and readmission risk.

Clinical management should align with patient-centered goals or those of surrogate decision-makers, informed by trajectories of functional recovery and overall prognosis [27], with high-risk individuals proactively identified and supported through individualized care plans—potentially enhanced by AI-enabled decision-support tools [28,29].

Following ICU discharge, care should encompass structured follow-up, rehabilitation programs targeting muscle strength, balance, and activities of daily living, optimization of chronic disease management, convalescent care for residual effects of acute illness, simple remote monitoring, and integration of community-based support services [30,31].

For geriatric patients with complex conditions, especially those at end-of-life, care must expect beyond the ICU to include robust community healthcare services. This necessitates strengthened support from RHM, nurses with specialized geriatric training, and comprehensive community services. In China, the central government had issued a series of policies to promote the construction of an integrated health service system that unifies the management and delivery of medical and health services across the continuum, including health promotion, disease prevention, treatment and hospice care. These initiatives aim to coordinate all levels of healthcare institutions according to patient needs, ensuring consistent, lifelong care.

## Doctor–patient shared decision-making (SDM)

Promoting SDM between clinicians and patients enhances patient engagement throughout the treatment process. This involves patients actively participating in medical decisions, fostering comprehensive communication between doctors and patients regarding the advantages and disadvantages of various treatment options.

Patient-specific factors such as preferences, socio-cultural background, education level, and economic status are systematically integrated, resulting in a collaborative decision-making process between clinicians and patients. Through SDM, patients are supported in selecting treatment options that align with both clinical evidence and their personal values.

However, in the ICU setting, many older patients are unconscious or cognitively impaired, necessitating SDM with legally designated surrogates or family members using advance directives, previously expressed wishes, or best-interest standards. Given that delirium and decision-making capacity can fluctuate over time, repeated assessments and timely re-engagement of geriatric patients are essential once attention and comprehension are restored [27,32].

The use of time-limited trials (TLTs) is recommended in cases of clinical uncertainty [27]. A TLT involves an agreement between clinicians and patients or their families to administer specific therapies for a defined period while monitoring the patient’s response against predetermined clinical outcomes. If improvement occurs, treatment continues with a disease-focused approach; conversely, if deterioration is observed, therapies are discontinued, often transitioning from curative to palliative care. Persistent uncertainty may warrant renegotiation of another trial [33].

Finally, within the framework of SDM, AI can streamline medical documentation, improve illness understanding among patients and surrogates, support comprehensive risk-benefit analyses, and provide basic psychological support, thereby facilitating a more informed and patient-centered decision-making process [34].

## Enhanced recovery for surgical or critically illness cases

Given the high prevalence of comorbidities in older adults and the clinical objectives of managing disease progression or alleviating symptoms, it is essential to implement early rehabilitation interventions aimed at mitigating complications such as delirium, venous thromboembolism, sarcopenia, and bone loss.

Geriatric interdisciplinary teams play a critical role in supporting rapid recovery from acute and critical illnesses through Acute Care for Elders (ACE) models or in promoting functional independence and self-management *via* Comprehensive Geriatric Assessment and Management (GEM) [35]. Co-management by geriatricians and surgical teams can reduce hospital length of stay and perioperative complications through standardized preoperative evaluations, expedited surgical procedures, and optimized postoperative care [36].

These interventions are closely aligned with key patient-centered outcomes in geriatrics, including cognitive function, quality of life, and long-term survival. Furthermore, emerging applications of AI and machine learning show considerable promise in enhancing the prediction and management of these comorbidities [37,38].

## Frailty assessment and early identification of high-risk older patients

All older adults with critical illness should undergo frailty assessment upon ICU admission [27]. The Clinical Frailty Scale (CFS) is the most widely reported tool for frailty assessment in the ICU [39]. However, the CFS has limitations compared to other instruments such as the Frailty Index (FI). While convenient to use, it lacks the comprehensiveness needed to capture conditions like depression, anxiety, or heart failure [40]. In contrast, the FI offers a more comprehensive evaluation of frailty and is better suited for repeated assessments over time.

Looking ahead, emerging tools such as the electronic Frailty Index (eFI) enable more detailed and real-time monitoring of frailty, supporting dynamic treatment adjustments in the ICU setting [41]. Moreover, AI is introducing novel capabilities into frailty assessment: AI-enabled standardization of CFS scoring can assist ICU clinicians without specialized geriatric training in rapidly and consistently obtaining reliable assessments [42].

Frailty assessment in geriatric patients requires multidisciplinary collaboration, particularly between intensivists and geriatricians. Upon ICU admission, intensivists focus on immediate life support and stabilization of the patient’s condition, while geriatricians conduct frailty assessments to identify high-risk individuals and inform personalized treatment plans aligned with patient values and overall health status.

As clinical stabilization is achieved, care transitions to a collaborative model in which intensivists and geriatricians jointly assess frailty, balance the intensity of interventions according to prognosis, manage acute medical issues, and adjust life-sustaining therapies as appropriate. After the acute phase, geriatricians assume primary responsibility for developing rehabilitation and chronic disease management strategies [27], while intensivists continue to manage residual acute complications during recovery.

## Conclusions

The adoption of new technologies in geriatric health monitoring is essential to effectively address the multifaceted challenges associated with critical illness in older adults.

Implementing the ABCDEF framework – comprising AI, Value-Based Medicine, Comprehensive Care Planning, Shared Decision-Making between Clinicians and Patients, Enhanced Recovery for Surgical or Critically Ill Patients, and Frailty Assessment and Early Identification of High-Risk Older Adults – can offer significant clinical and operational benefits. By integrating this framework, healthcare providers can deliver more compassionate, competent, and individualized care, thereby bridging the gap between research and clinical practice. Ultimately, this structured approach has the potential to improve patient-centered outcomes while enhancing the efficiency and effectiveness of geriatric critical care systems.

## Data Availability

Data sharing is not applicable to this article as no data were created or analysed in this study.
